# The Case of Moulay Ismael - Fact or Fancy?

**DOI:** 10.1371/journal.pone.0085292

**Published:** 2014-02-14

**Authors:** Elisabeth Oberzaucher, Karl Grammer

**Affiliations:** Department of Anthropology, University of Vienna, Vienna, Austria; Hungarian Academy of Sciences, Hungary

## Abstract

Textbooks on evolutionary psychology and biology cite the case of the Sharifian Emperor of Morocco, Moulay Ismael the Bloodthirsty (1672–1727) who was supposed to have sired 888 children. This example for male reproduction has been challenged and led to a still unresolved discussion. The scientific debate is shaped by assumptions about reproductive constraints which cannot be tested directly—and the figures used are sometimes arbitrary. Therefore we developed a computer simulation which tests how many copulations per day were necessary to reach the reported reproductive outcome. We based our calculations on a report dating 1704, thus computing whether it was possible to have 600 sons in a reproductive timespan of 32 years. The algorithm is based on three different models of conception and different social and biological constraints. In the first model we used a random mating pool with unrestricted access to females. In the second model we used a restricted harem pool. The results indicate that Moulay Ismael could have achieved this high reproductive success. A comparison of the three conception models highlights the necessity to consider female sexual habits when assessing fertility across the cycle. We also show that the harem size needed is far smaller than the reported numbers.

## Introduction

The scientific discussion about the case of the Sharifian Emperor of Morocco, Moulay Ismael the Bloodthirsty (1672–1727), who is reported to have sired 888 children in the Guinness Book of Records, is still ongoing. Recent findings on the genetic legacy of Genghis Khan and his male relatives show impressively how vast the distribution of genes of powerful males can be: About 8% of todays' Asian male population dates back to this family [1]. Einon [2] claimed that it is unlikely that Moulay Ismael could have had that many offspring, based on several factors potentially affecting reproduction: Ovulation frequency combined with sperm survival (resulting in women being fertile only 12.5% of the time), female infertility (8%), conception likelihood (58%), and prenatal mortality (15–20%). Contrarily, Gould [3] states that Einon's assumptions are unrealistic insofar, as some assumptions were not correct: First, Einon inaccurately used Moulay's duration of reign as life span, but also the biological parameters were flawed: Sperm survival is about twice as long as Einon posed [4], and fertility of cycles in women between 25 and 39 is as high as 93–98% [5]. The likelihood of foetal loss is highest in the first 14 days of pregnancy [6], after the first 14 days of pregnancy (which corresponds to the data basis of Jöchle) is around 5% [7], child mortality until the age of two around 20% [8], [9], [10]. To complete the picture, sperm aging and decline of sperm vitality have to be taken into account [11], [12].

One problem lies in the historical facts. Fortunately, there is a report by Dominique Busnot [13], a French diplomat, who was in Morocco in 1704, 1708 and 1712. We decided to use his report in the year 1704 as the data source for our calculations, as it provides figures not only about the number of offspring, but also about reproductive timespan. Moulay became emperor in 1672 at age 25 and was thus 57 when Busnot first visited Morocco. In 1704 Busnot reports Moulay to have 600 sons from four wives and 500 concubines. Daughters by his four wives were allowed to live, whereas daughters born by his concubines were suffocated by the midwifes at birth. This results in approximately 1171 children from 500 women in a reproductive time span of 32 years (25–57). Note that we neglect the reproduction before his becoming emperor, as he most likely did not have a comparable harem then. Moulay Ismael took extreme measures to ensure paternity security, which is partially responsible for his sobriquet “the bloodthirsty”. Any suspicion of adultery was severely punished: The women were either strangled by himself, or their breasts were cut off, or their teeth were torn out. This applied even to former concubines who had already left the harem - which they had to when reaching the age of 30. Men who merely looked at one of his wives or concubines were punished by death penalty [13].

As this report of Busnot appears to be the only reliable source of information available, we focus on the reproductive data of the 1704 report, rather than estimating his life-time reproductive success. Since Moulay died in 1727 it is likely that those numbers could have been substantially larger.

Calculations of reproductive effort have to include a number of interacting variables, and thus cannot be done sequentially, but have to take the dynamic nature of a reproduction pool into account. Thus, we decided to model Moulay Ismael's reproductive efforts in a computer simulation.

There are two conception models that are widely used to assess the conception likelihood across the female cycle: The Wilcox-Weinberg model [14] and the Barrett-Marshall [15] model. Both models are based on data from couples participating in longitudinal studies. The former participated in a general medical study, whereas the latter used the temperature method for birth control. In both cases the women had born at least one child before participation. The conception model by Jöchle [16] differs greatly from those two, not only regarding the nature of the data, but in the distribution of conception likelihood over the cycle. Jöchle's model is based on data from German soldiers' wives during the two world wars, who had intercourse with their husbands only when they were transferred from one front to the other, and on conceptions resulting from rape. Jöchle concluded from this data that under specific conditions induced ovulation might happen in humans: Rare sexual intercourse and strong emotional arousal [16]. It is likely that these conditions would have held true for the concubines of Moulay Ismael. Sperm potency seems to be unaffected by frequent intercourse [17].

## Methods

**Figure 1:**
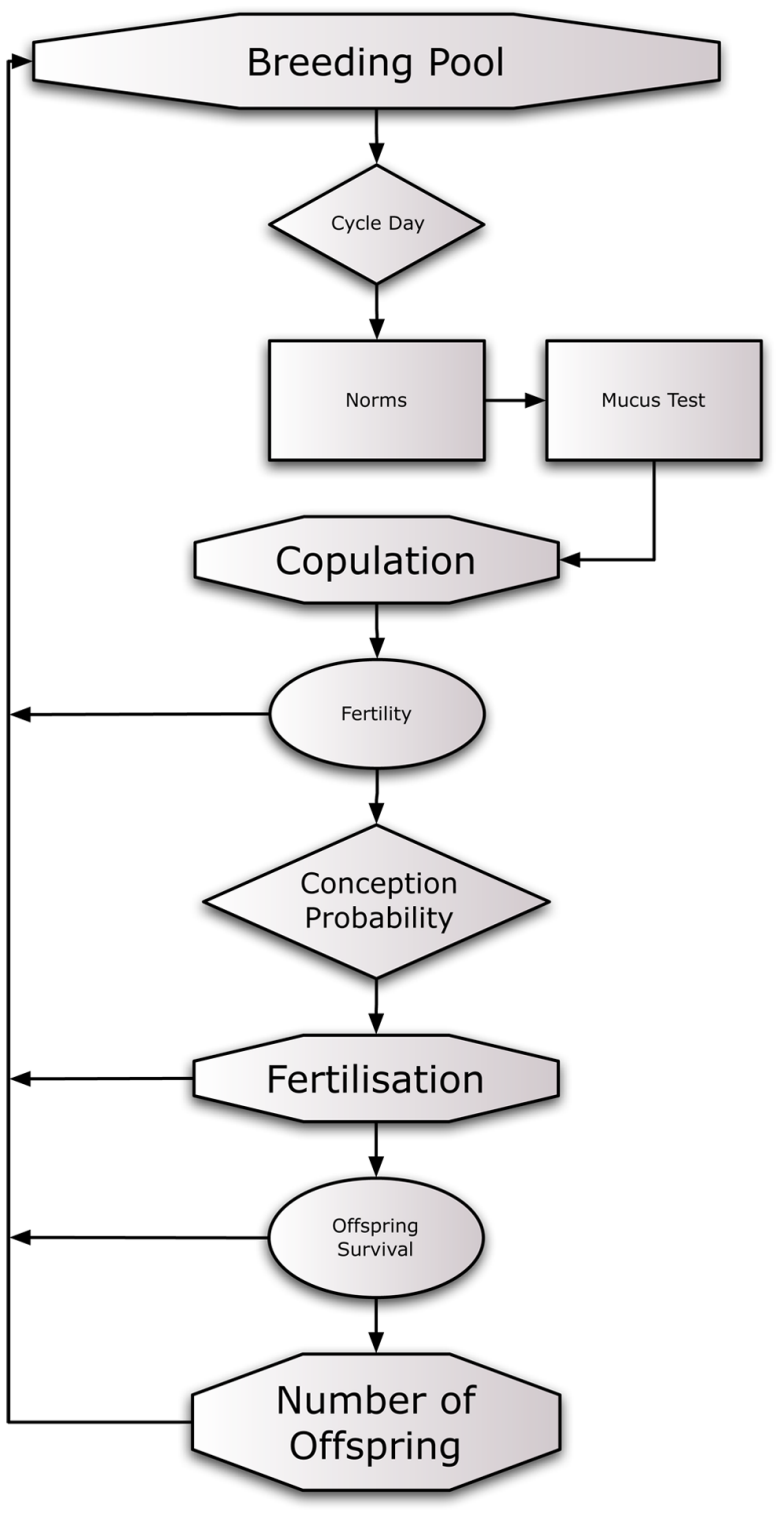
This figure illustrates the setup of the simulation—circles indicate factors negatively affecting reproductive success, rectangles indicate factors increasing reproductive success.

We used Python for the development of the simulation. The source code is available as Script S1. For both simulations we used the following three models as a basis: a) the Wilcox-Weinberg Model of Conception [14], which integrates observational data with the survival rate of sperm and the viability of the cycle, b) the conception likelihood of Jöchle [16], and the Barrett-Marshall model [15]. These basic models were integrated in our simulation, in which we incorporated potential constraints (Fig. 1).

The breeding pool consists of 504 women (500 concubines plus 4 wives), the cycle day was assigned randomly in the first simulation, in the second we allowed for 50% synchronisation, i.e. 50% of the women would cycle simultaneously. This value was chosen completely arbitrarily for the purpose of investigating whether this would have an effect at all. Cycle synchronization is still much disputed [18], [19], [20]. Cultural norms stand for the copulation taboo during menstruation, i.e. five days of the period of the female cycle with lowest fertility [21]. The mucus test allows to detect ovulation ±4 days with an accuracy of 97% [22], but as it is unknown whether it was practiced in Morocco at that time, we chose to base our simulation on behavioural data: Ovulatory faces are perceived as most attractive in a forced choice task at 60% [23], which aligns well with other studies on attractiveness over the female cycle [24], [25]. Another advantage is that we do not need to assume detailed knowledge about fertility and menstrual cycle, but base our calculations on mere increased sexual attraction in the most fertile period. Copulation leads to conception depending on the viability of sperm, with a decrease of 1.52% per year [11], [12], fertility of the female's cycle [5], and on the cycle day [14], [15], [16]. Taking all these constraints into account, we calculate the conception probability leading to fertilisation as detected through observation, i.e. absence of monthly bleeding. Once fertilisation has been detected (after day 14 of pregnancy), foetal loss [7] and offspring survival rate [8], [9], [10] again reduce the number of offspring.

In the first model we calculated how many copulations per day would be necessary to reach 1171 offspring in 32 years, and how the constraints delineated above modulate this number. In the second model we calculated the number of offspring reached in the reproductive period of 32 years and how the variables affect this number. For these models we calculated 200 iterations.

We reran the simulations four times, once with ovulation detection and without sperm ageing, once without ovulation detection and sperm ageing, once with both, ovulation detection and sperm ageing, and once with sperm ageing and without ovulation detection.

Lastly, we calculated how the harem size affects the calculations. Based on the restrictions used in model number one, we calculated the number of offspring for harem sizes between 1 and 200. Pregnant women were removed from the reproductive pool for a period of 18 months, allowing for pregnancy and lactation [26].

## Results

### How many copulations a day must a man have?

In the first step we simulated random access to the harem pool and calculated the number of copulations per day which were necessary to reach the given number of children. In subsequent steps we added constraints to the model to investigate their effect on the reproductive effort needed.

The constraints we used were religious taboos (no copulations for five days each cycle during menstruation), the possibility of ovulation detection (with an accuracy of 0.6, and foetal and child mortality. If copulations happened completely random, 1.97 (Wilcox), 0.83 (Jöchle) or 2.30 (Barrett-Marshall) copulations per day would have lead to a reproductive success of 1171 offspring. Cultural norms and ovulation detection decrease the required number of copulations substantially, while foetal loss and child mortality increase it. Taking all constraints into account leads to an average of 1.43 (Wilcox), 0.83 (Jöchle) or 1.63 (Barrett-Marshall) copulations per day. (see Table 1) All constraints used in the model have a significant effect on the required reproductive effort (t-test, p<0.001).

**Table 1:** Model I: Copulations per Day required reaching 1171 offspring.

	Wilcox	Jöchle	Barrett-Marshall
Constraints	Copulations per Day (min-max)	Copulations per Day (min-max)	Copulations per Day (min-max)
Random	1.97 (1.83–2.13)	0.83 (0.78–0.88)	2.30 (2.14–2.45)
Cultural Norms	1.59 (1.50–1.75)	0.75 (0.70–0.80)	1.87 (1.76–1.99)
Ovulation Detection	1.27 (1.19–1.37)	0.68 (0.64–0.73)	1.44 (1.35–1.55)
Foetal Loss	2.08 (1.94–2.22)	0.87 (0.81–0.93)	2.43 (2.27–2.62)
Child Mortality	2.46 (2.30–2.66)	1.03 (0.96–1.10)	2.87 (2.71–3.06)
All Constraints	1.43 (1.33–1.52)	0.83 (0.78–0.88)	1.63 (1.53–1.73)

200 simulations were iterated until 1171 children were reached. Female cycle day was randomly assigned.

### How many children can one man have?

In the second model we calculated the number of children which could be sired given one copulation a day throughout the reproductive period of 32 years. First, we calculated the number of offspring when copulating on a random basis. Then we calculated the effect of intervening variables. Besides the constraints of model one we included the possibility of ovulation synchronisation and the emergence of love and favouritism. In this simulation calculations with all three models indicate that the number of offspring could have been reached. Results indicate that with only one copulation per day, Moulay could have succeeded in siring the fabled number of offspring only when calculations are made based on the Jöchle model. In the simulation based on the Wilcox-Weinberg model Moulay it depends very much on the constraints included in the simulation, whether 1171 offspring can be achieved or not. Especially when taking into account the decrease of sperm quality with age, ovulation detection becomes crucial for reproductive outcome. The results based on the Barett-Marshall model indicate that it was impossible to achieve the reported number of offspring without ovulation detection given only one copulation per day. (Table 2).

**Table 2:** Model II: Number of Children/Reproductive Span based on one copulation per day.

	Wilcox	Jöchle	Barrett-Marshall
Constraints	Number of children (min-max)	Number of children (min-max)	Number of children (min-max)
No Constraints	583 (520–629)	1380 (1309–1460)	502 (452–556)
Basic Constraints: Cultural Norms, Ovulation Detection, Foetal Loss & Child Mortality			
Basic Constraints Only	802 (748–858)	1387 (1315–1460)	713 (665–765)
Ovulation Sync.	735 (686–780)	1323 (1249–1381)	650 (608–691)
Moulay Falls in Love	556 (515–604)	903 (851–955)	491 (451–525)
Favourites	805 (753–854)	1386 (1309–1460)	704 (649–754)
All Constraints	535 (496–589)	892 (830–946)	467 (424–506)
Basic Constraints: Cultural Norms, Fetal Loss & Child Mortality			
Basic Constraints Only	556 (502–625)	1164 (1088–1220)	475 (445–514)
Ovulation Sync.	555 (504–609)	1159 (1100–1226)	477 (439–523)
Love	445 (408–487)	815 (771–855)	390 (343–422)
Favourites	552 (511–599)	1164 (1097–1233)	479 (438–523)
All Constraints	446 (405–499)	826 (773–883)	392 (350–440)
Basic Constraints: Cultural Norms, Ovulation Detection, Sperm Aging, Fetal Loss & Child Mortality			
Sperm Aging Only	450 (398–497)	1060 (994–1119)	385 (342–432)
Basic Constraints Only	617 (565–659)	1066 (989–1134)	547 (492–600)
Ovulation Sync.	569 (526–604)	1014 (946–1087)	499 (449–542)
Love	437 (396–478)	717 (672–768)	386 (339–422)
Favourites	620 (557–659)	1060 (997–1127)	540 (480–591)
All Constraints	424 (391–459)	708 (657–759)	367 (324–401)
Basic Constraints: Cultural Norms, Sperm Aging, Fetal Loss & Child Mortality			
Basic Constraints Only	415 (364–457)	898 (824–955)	367 (333–402)
Ovulation Sync.	427 (383–481)	896 (826–947)	364 (330–402)
Love	357 (323–397)	654 (616–694)	309 (278–346)
Favourites	421 (386–465)	896 (843–958)	366 (330–404)
All Constraints	356 (327–388)	656 (619–691)	311 (270–353)

In 100 simulations we modelled the complete life span reproductive success based on one copulation a day. Basic constraints are female cycle, harem size, reproductive span, female age, pregnancy and lactation. According to Muslim customs a harem owner can have four wives and eleven favourites.

All constraints used in the model have a significant effect on the number of offspring (t-test, p<0.001).

### How many women does one man need?

**Figure 2:**
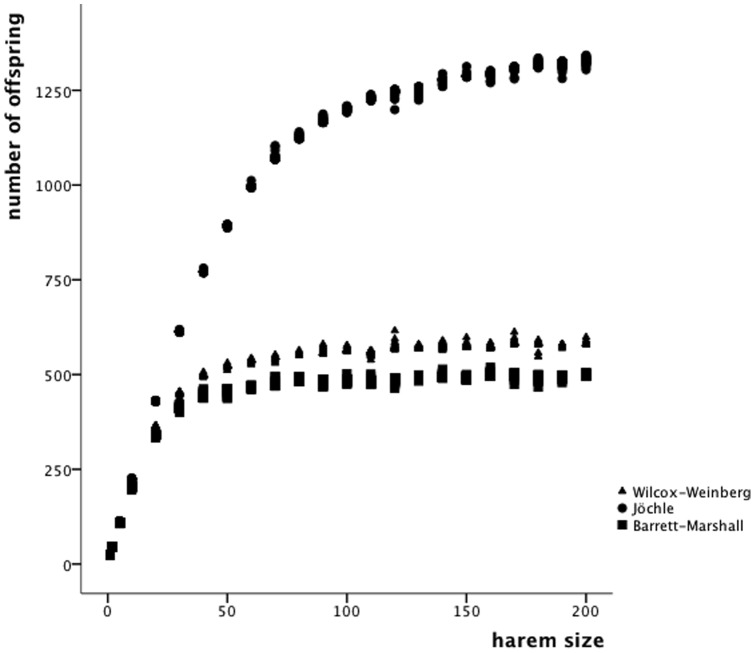
The potential reproductive outcome is related to the harem size. Saturation is reached at smaller harem sizes for the Wilcox-Weinberg and Barrett-Marshall models than for the Jöchle model.

Our calculations indicate that the harem size necessary to reach the reproductive outcome of 1171 children is far lower than the reported 504. The number of offspring reaches saturation at much smaller breeding pools. Calculations based on the conception model by Jöchle indicate that a harem size beyond 110 does not lead to an increased number of offspring (Quadratic regression: R^2^ = .978, df = 2274, p<0.001; Y = 86.262−0.057x^2^+17.041x). For the Wilcox-Weinberg model the saturation is reached at a harem size of about 70 (Quadratic regression: R^2^ = .864, df = 2274, p<0.001; Y = 133.701−0.028x^2^+7.436x), and for the Barrett-Marshall model reproductive outcome does not increase beyond a harem size of 65 (Quadratic regression: R^2^ = .835, df = 2274, p<0.001; Y = 131.665−0.024x^2^+6.241x). (Fig. 2)

## Discussion

In general, results indicate that the Emperor could have reached his notorious reproductive success with fewer copulations than assumed so far - thus the historic reports could be facts and not fancy. With our simulation we could also provide evidence that the harem size is of lesser importance for the achievement of the reported reproductive success than thought so far. A breeding pool of 65 to 110 women leads to the maximum reproductive outcome. This highlights the importance of incorporating cost-benefit calculations – increasing the size of the breeding pool beyond that point increases the costs without additional benefits to outweigh them. Having a harem of 500 concubines might have been due to other considerations than maximization of individual reproductive outcome. For example, it could have been a means to remove the additional women from the reach of other men, thus depriving them of reproductive potential.

We also show that the choice of conception model has to be carefully considered. Sexual habits have a strong impact on the distribution of conception likelihood over the female cycle. Therefore it is essential to take frequency of intercourse into account when trying to estimate the likelihood of pregnancy resulting from intercourse. In our case, the sexual habits of the concubines were most likely similar to the sexual habits of women in the Jöchle databases, i.e. rare intercourse due to the large number of women in the harem.

In our models, we intentionally chose to incorporate more conservative assumptions about the effect sizes of the involved variables (i.e. foetal loss, child mortality, …). This means, that we always chose the figure most adverse to number of offspring. As the goal was to investigate whether the historic reports about the reproductive success of Moulay Ismael can be correct, rather than estimating the maximum number of offspring possible for a man, this was the method of choice. When addressing different scientific questions, one might choose to change these figures.

Besides contributing to the dispute about the limits of male potential reproductive success by shedding light on the most popular example in this debate, this study also provides a rationale for the choice of conception models. While female sexual habits have not been considered so far, this study emphasizes the importance to take them into account.

## Supporting Information

Script S1 **The Python script we used for the simulation.** Comments in the program explain the variables used and describe the steps of the calculations.
(PY) Click here for additional data file.
